# Cyclin A2 and Ki-67 proliferation markers could be used to identify tumors with poor prognosis in African American women with breast cancer

**DOI:** 10.46439/cancerbiology.4.048

**Published:** 2023

**Authors:** Desta Beyene, Tammey Naab, Victor Apprey, Luisel Ricks-Santi, Ashwini Esnakula, Mustafa Qasim, Mathew George, Karen G Minoza, Robert L Copeland, Carolyn Broome, Yasmine Kanaan

**Affiliations:** 1 Howard University Cancer Center, Washington, DC 20060, U.S.A.; 2 Pathology Department, Howard University Hospital, Washington, DC 20060, U.S.A.; 3 Department of Pharmacotherapy and Translational Research, College of Medicine, University of Florida, Gainesville, FL 32610, U.S.A.; 4 Pathology Department, James Comprehensive Cancer Center, Columbus, OH 43210, U.S.A.; 5 Department of Microbiology, Howard University College of Medicine, Washington, DC 20059, U.S.A.; 6 Department of Biochemistry, Howard University, College of Medicine, Washing, DC 20059, U.S.A.; 7 Division of Trauma, Critical Care and Acute Care Surgery, Department of Surgery, Oregon Health & Science University, Portland, OR 97239, U.S.A.; 8 Department of Pharmacology, Howard University College of Medicine, Washington, DC 20059, U.S.A.

**Keywords:** African American, Breast cancer, Protein markers, Cluster analysis, Breast cancer subtypes

## Abstract

**Background:**

Diagnosed invasive breast carcinomas in African American patients are more aggressive compared with those in Caucasian patients and diagnosed at later stages of the disease with higher grade tumors. Despite advances in breast cancer systemic treatment, new prognostic and predictive biomarkers are still needed. Therefore, potential biomarkers were chosen to correlate with different subtypes, recurrence, and survival of invasive breast cancer in a cohort of African American women.

**Methods:**

Eight protein biomarkers (ER, PR, HER2, Cyclin A2, Cytokeratin 5, Vimentin, Bcl2, and Ki-67) were evaluated using tissue microarrays (TMAs) and immunohistochemistry (IHC). The IHC results from TMAs were analyzed by both supervised and unsupervised clustering methods. The predictive clusters for the supervised and unsupervised methods were compared for agreement with the empirical classification. Kappa values were used to determine the overall percent correct clusters and agreement between specific clusters. Chi-square statistics was used to examine the association between hierarchical and multinomial logistic clustering methods.

**Results:**

Five subtypes of breast tumors with distinct protein expression patterns were identified among the studied 166 breast tumors. Luminal B tumors have been distinguished from luminal A tumors by staining for cell cycle proteins Cyclin A2 and Ki-67, which promote cell proliferation. Forty-nine percent were stained positive for Cyclin A2, 39.2% positive for Ki-67, and 37% positive for both Cyclin A2 and Ki-67. The age of patients did not show any significant effect whether five (p-value= 0.576) or eight (p-value= 0.605) biomarkers were used, which indicating that age did not have any influence on the classification of the subtypes. Ninety percent of the thirty triple negative tumors were positive for Cyclin A2 or Ki-67 or both. Six-year overall survival was better for luminal A tumors (76%) than luminal B tumors (71%). Likewise, six-year relapse-free survival was better for luminal A tumors (76%) than luminal B tumors (29%).

**Conclusion:**

Discovery of molecular markers such as Cyclin A2 and Ki-67, and subtypes that are most prevalent in African Americans could lead to a better understanding of the factors contributing to higher morbidity and mortality in this group and to aid in decision-making to offer earlier treatment.

## Introduction

Invasive breast carcinoma is the most diagnosed cancer in women in all racial groups in the United States [1]. It is estimated that about 287,850 new cases of invasive breast cancer will be diagnosed in women during 2022 [2]. Breast carcinoma is the leading cause of cancer mortality in African American women [3]. Over the last decades, a paradoxical correlation has been observed among African American women in terms of higher breast cancer mortality rates despite a lower incidence of breast cancer diagnoses compared with Caucasian women [4–6]. A greater proportion of breast cancers are diagnosed in African American women of younger age groups compared with Caucasian women (33% of African American women age<50 years vs 25% of Caucasian women) [7,8]. Invasive breast carcinomas diagnosed in African American patients are described as more aggressive compared with those in Caucasian patients and diagnosed at later stages of the disease with higher grade tumors [9,10]. Furthermore, African American women are more likely to have hormone receptive-negative breast cancers than Hispanic women, non-Hispanic White women, and Asian women [11,12]. Moreover, African American cancer patients have shorter survival compared to White women [13].

The recent trend toward improvement in breast cancer mortality rate is due to increased diagnosis of early-stage diseases. Newly developed techniques such as DNA microarrays and Serial Analysis of Gene Expression (SAGE) have helped to analyze molecular differences between normal and cancer cells [14–16]. These approaches have enabled us to classify breast cancer at a molecular level and identify molecular signatures that correlate with metastatic behavior and clinical outcome. Genomic analyses of breast cancer phenotypes have sub-classified breast cancers into four important categories, each with different clinical behavior [17–19]. These include the luminal A [Estrogen-positive (ER^+^) and/or Progesterone-positive (PR^+^), Human Epidermal Growth Factor Receptor 2-negative (HER-2^−^)]; Luminal B (ER^+^, and/or PR^+^, HER-2^+^); HER-2 enriched [ER-negative (ER^−^), PR-negative (PR^−^), HER-2-positive (HER-2^+^)]; and Basal-like (ER^−^, PR^−^, HER-2^−^, Cytokeratin 5/6+ (CK5/6), and/or HER-1^+^) phenotypes. On the other hand, recent DNA microarray profiling studies on breast tumors have identified five distinct subtypes of breast carcinomas [16,17,20–22]: Basal-like [triple-negative (ER^−^, PR^−^, HER-2^−^), CK5/6^+^ and/or HER-1^+^]; Basal-like/ luminal B (HER-2^+^, ER^−^, PR^−^); Luminal B (HER-2^+^, ER^+^ and/or PR^+^); Luminal A (HER-2^−^ , ER^+^ and/or PR^+^); and unclassified subtype (tumors that are negative for ER, PR, HER-2, HER-1, and CK5/6).

Several investigators have used tissue microarrays [TMAs] to study the expression of different proteins in breast tissue and other tissues [23–25]. TMAs also has been used to identify and/or validate molecular signatures in breast cancer [26–28]. TMA immunohistochemistry [IHC] is less expensive, time saving and routinely used in clinical laboratories. Many researchers have used data from TMA of breast cancer samples to correlate with prognosis [21,25,29–33]. TMA identifies the signature profiles of multiple proteins that are useful for early detection of the primary cancer and recurrence, prediction of survival, identification of tumor subtypes, and drug design. Different scoring systems exist for semi-quantitative evaluation of immunohistochemical staining of breast cancer tissue. Most widely used systems to determine the levels of nuclear, cytoplasmic, and membrane staining in different proteins are the Diallo score [34], Alfred score [35], Histochemical score (H-score) [36] and Quick score [37]. Despite advances in breast cancer systemic treatment, new prognostic and predictive factors are still needed; and the correlation of protein biomarkers with breast cancer subtypes in African Americans could lead to a better understanding of the factors that may be contributing to mortality, prognosis, treatment, and targets for therapy. Therefore, the purpose of this study was to characterize protein markers in African American breast tumors that correspond to different subtypes, recurrence, and survival.

## Materials and Methods

### Breast tissue samples

A total of 166 formalin-fixed paraffin-embedded (FFPE) breast tissue blocks were retrieved from the Pathology Department at Howard University Hospital with the approval of Howard University Institutional Review Board (IRB) that correspond to different tumor subtypes, recurrence, and survival to characterize protein markers in African American breast cancer tumors. The African American breast cancer patients were diagnosed with an invasive breast ductal carcinomas (IDC) at Howard University Hospital between 2002 and 2009.

### Tissue microarrays (TMAs) construction

Triplicate TMAs were constructed in our lab as described by Jacquemier Jocelyne [32], Diallo-Dane Brock [34], and Hewitt SM [38]. Five-micron sections were cut from the archived FFPE blocks, and float mounted on super frost plus micro slides. Slides were stained with hematoxylin-eosin (H&E) and representative areas with IDC were identified and marked on the H&E slides by the pathologist. The individual marked slides were placed on top of each donor block (archived/original FFPE block) and carefully aligned to locate the corresponding tumor sites where the core samples are to be collected. Three separate 1.0 mm tissue cores were obtained from each FFPE donor block and mounted in triplicate recipient TMA blocks using Automated Tissue Arrayer ATA-27 (Beecher Instruments Inc., Pathological Devices, Wisconsin, USA). Spacing between adjacent cores in the recipient TMA blocks was 3 mm. The TMA blocks with core samples were tempered by incubation overnight at 37 °C. Using a microtome, 5-μm sections were cut from the TMA blocks and mounted on Superfrost Plus microscope slides (Thermo Fisher Scientific, MA, USA) for IHC staining.

### Immunohistochemical (IHC) staining

The IHC markers were selected based on those known to differentiate breast cancer subtypes with cDNA microarrays, TMA, and protein staining [20,31,36–38]. TMA sections were immune-stained with monoclonal antibodies specific for ER, PR, HER-2, CK5, Vimentin, B-cell lymphoma 2 (Bcl2), Cyclin A2 and Ki-67 (Table 1). The TMA slides were deparaffinized in xylene twice for 5 minutes each, rehydrated with a graded series of alcohol 100%, 95% and 70% ethanol [vol/vol] in deionized water for 5 minutes each. Antigen retrieval for the above selected markers was performed by incubating the slides in Invitrogen 0.01 M citrate buffer (pH 6.0) in a 100°C vegetable steamer (Oster, Sunbeam Products Inc., Boca Raton, Florida, USA) for 20 minutes. Slides were rinsed with phosphate buffer saline (PBS, pH 7.5) at the end of each step in the procedure. Endogenous peroxidase activity was quenched by incubation in 3% hydrogen peroxide solution with shaking for 15 minutes at room temperature. Sections were then incubated in unlabeled blocking 1.0% normal goat serum (Vector Laboratories, Burlingame, CA, USA) for 30 minutes at room temperature. After that, the slides were incubated at 4°C overnight in a humidified chamber with one of the monoclonal antibodies as described in Table 1. The dilution of each antibody was established based on negative and positive controls and staining with a range of dilutions (Table 1). The slides were incubated with the appropriate primary antibody and then washed with PBS (pH 7.0) containing 0.1% triton and 0.1% bovine serum albumin. After 30 minutes of incubation at room temperature with a biotinylated goat anti-mouse IgG (0.5%)/ streptavidin complex (VWR, PA, USA), and the positive reaction was visualized using the Avidin Biotin Complex method (ABC kit, Vector Lab). The chromogen substrate was diaminobenzidine (DAB kit, Invitrogen, Waltham, MA, USA). Stained slides were counterstained with hematoxylin (Invitrogen) and finally treated with 70%, 95% and 100% ethanol and xylene. Slides were cover slipped with an automatic unit (Tissue-Tek SCA, Thermo Fisher Scientific) and observed under the light microscope.

### Evaluation of immunohistochemical staining

Stained TMA slides were evaluated based on proportion score (%) and relative staining intensity by two pathologists as described by Diallo-Dane Brock [34] and Aaltonen et al. [39]. Slide scores on the bases of proportion of cells stained (extent) and relative staining intensity in the invasive tumor cells present in the tissue core were recorded in Excel file. The Diallo score [34] was used to determine the levels of staining for ER, PR, HER-2, Cyclin A2, Bcl2, CK5, and Vimentin markers. The Diallo scoring system used is as follows: Extent of % stained cells – 0 = no cells stained positive; 1 = <25% positive; 2 = 26–50% positive; 3 = 51–75% positive; and 4 = >75% positive. Intensity of stained cells: 0 = negative, 1 = weak staining; 2 = moderate staining; and 3 = strong staining. The intensity and proportion (extent) scores were multiplied to give a final score ranging from 0 to 12. A sample was judged to be negative with a score of 0 to 3 and positive with score of 4 to 12. The scoring system developed by Cheong et al. [40] and Veronese et al. [41] was used for Ki-67; Ki-67 was interpreted as being increased when >14% of cells show nuclear expression. Membranous IHC staining of +3 or greater for HER-2 was considered positive; +2 scores were re-evaluated by fluorescence in situ hybridization (FISH). Tissue cores that did not adhere to the slide, had no invasive carcinoma, or were otherwise uninterpretable were excluded from scoring and data analysis.

### Statistical analyses

Eight markers (ER, PR, HER-2, Cyclin A2, CK5, Vimentin, Bcl2 and Ki-67) were evaluated for identifying breast cancer subtypes in African American women using TMA and IHC. The TMA results were analyzed by both supervised and unsupervised clustering methods. For the unsupervised method, hierarchical clustering of the proteins and their distribution among the 166 FFPE samples were constructed using Cluster 3.0 and Java TreeView software Version 20 [42]. Hierarchical clustering, using complete linkage as cluster method and Euclidean as distance measure [43], was employed to classify samples based on all eight proteins. The NCSS software [44] was used to construct the dendrogram and to show the protein expression profile among the 166 tested breast cancer tumors.

For the supervised method, multinomial logistic regression analysis [45,46] was conducted using an empirically classified training data set. The empirical classification was based on previously published cDNA microarrays, reverse transcription-quantitative polymerase chain reaction (RT-qPCR), TMA, and protein studies [20,32,34,47,48]. The predictive clusters for the supervised and unsupervised methods were compared for agreement with the empirical classification. Kappa values [49] were used to determine the overall percent correct clusters and agreement between specific clusters. For high values of Kappa, the lower limit of the confidence interval was used for verification. Chi-square statistics [50] was used to examine the association between hierarchical and multinomial logistic clustering methods.

## Results

### TMA construction and IHC staining

**Figure 1.**
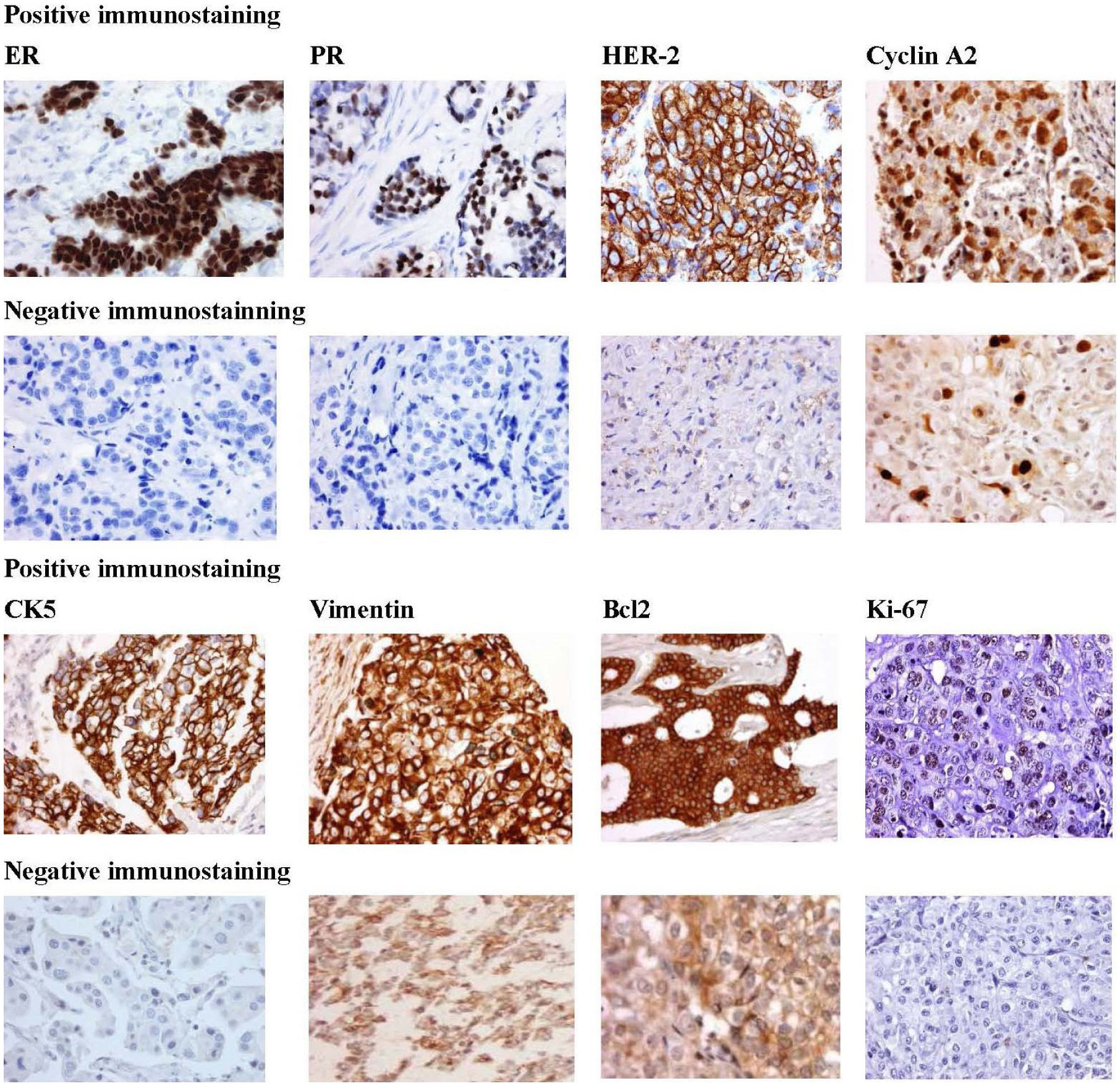
Representative immunostaining for the protein biomarkers [Estrogen receptor (ER), Progesterone receptor (PR), Human epidermal growth factor receptor 2 (HER2), Cyclin A2, Cytokeratin 5, Vimentin, Bcl2, and Ki-67] in African American breast tumors. Positive staining (rows 1 and 3) demonstrates strong staining of primary antibody. Negative staining (rows 2 and 4). Magnification is at 40X.

TMA have been constructed, prepared and stained for proteins markers (Figure 1) (ER, PR, HER-2, Cyclin A2, CK5, Vimentin, Bcl2, and Ki-67) that would distinguish 5 tumor subtypes: luminal A (ER^+^and/or PR^+^, HER-2^−^); luminal B (ER^+^and/or PR^+^ , HER-2^−^, Cyclin A2^+^ and/or Ki-67^+^); luminal B HER-2^+^ (ER^+^, and/or PR^+^, HER-2^+^); HER-2^+^ (ER^−^, PR^−^, HER-2^+^), and triple-negative (ER^−^, PR^−^, HER-2^−^). These subtypes are known to be associated with different relapse-free survival and overall survival.

### Biomarkers expression

Forty-nine percent were stained positive for cyclin A2, 39.2% positive for Ki-67, 55% positive for Bcl2, and 37% positive for both Cyclin A2 and Ki-67 (Table 2).

**Figure 2.**
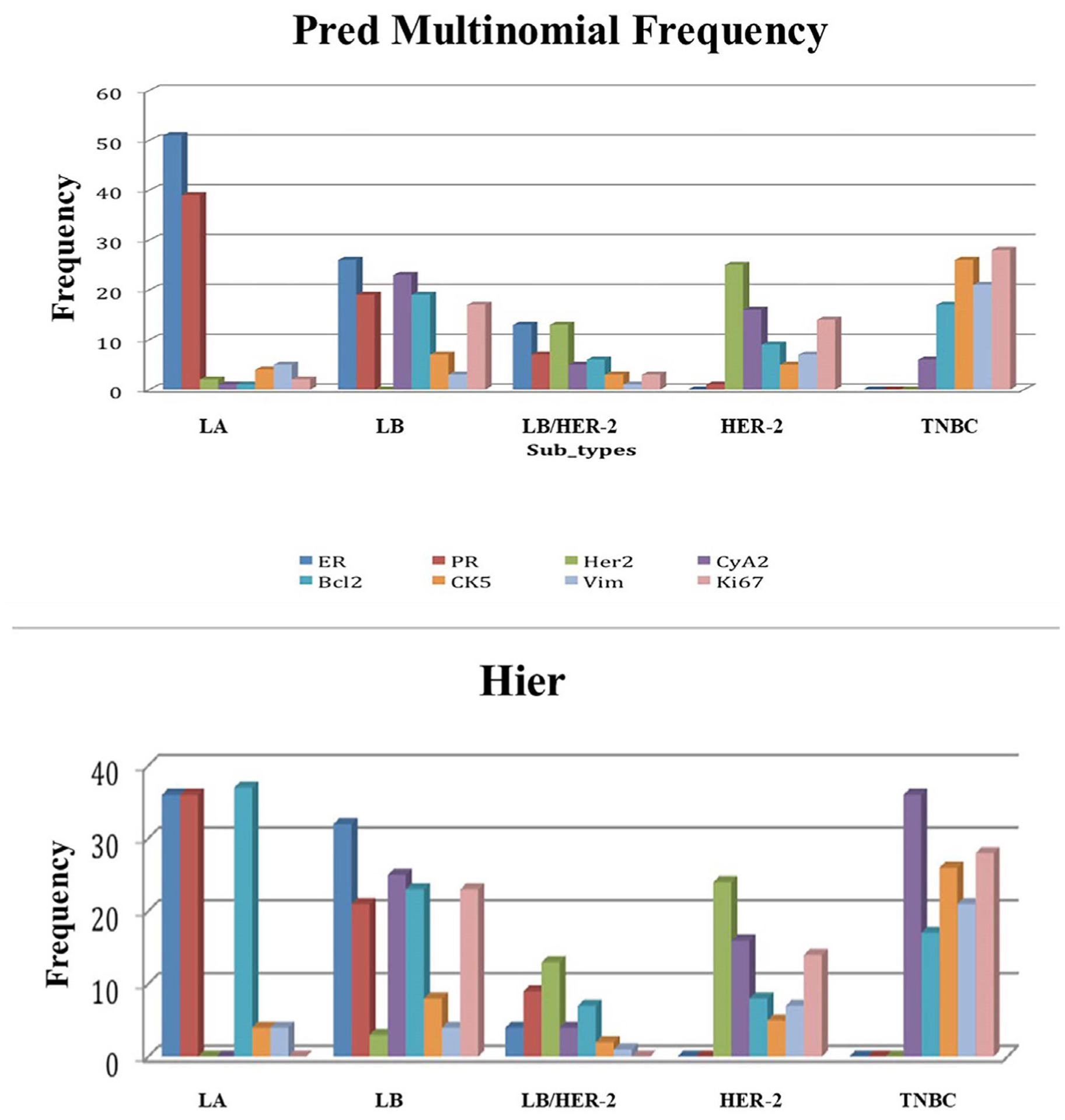
Predicted frequency of positively stained tumors within each subtype. The highest frequency of positive immune-reactivity for Cyclin A2 and Ki-67 was observed in the luminal B and triple-negative breast cancer subtypes. Whereas the lowest activity of these two biomarkers was in luminal A. **2a.** Predicted frequency according to multinomial logistic regression. **2b.** Predicted frequency according to hierarchical clustering. X-axis represents the 5 breast cancer subtypes. Y-axis represents the frequency of each of the 8 tested biomarkers in each breast cancer subtype. LA: Luminal A; LB: Luminal B; LB/HER-2: Luminal B/Human Epidermal growth factor Receptor 2; HER-2: Human Epidermal growth factor Receptor 2; TNBC: Triple Negative Breast Cancer; ER: Estrogen Receptor; PR: Progesterone Receptor; CyA2: Cyclin A2; Bcl2: B-cell lymphoma-2; Vim: Vimentin.

The distribution of the 166 breast cancer tumors among the different clusters is shown in Table 3. Most of the tumors belonged to luminal A (30.7%) and triple-negative (29.5%) clusters. The highest frequency of positive immuno-reactivity for Cyclin A2 and Ki-67 was observed in the luminal B and triple-negative subtypes (Figure 2). The lowest activity of these two markers was in luminal A. Whereas, the expression of Bcl2 was higher in luminal A than luminal B tumors. Therefore, prognostic role of Bcl2 expression in breast cancer is subtype-specific, and Bcl2 expression differs according to the molecular subtype and is a good prognostic marker for only luminal A breast cancer.

### Influence of age on the classification of the breast cancer subtypes

**Figure 3.**
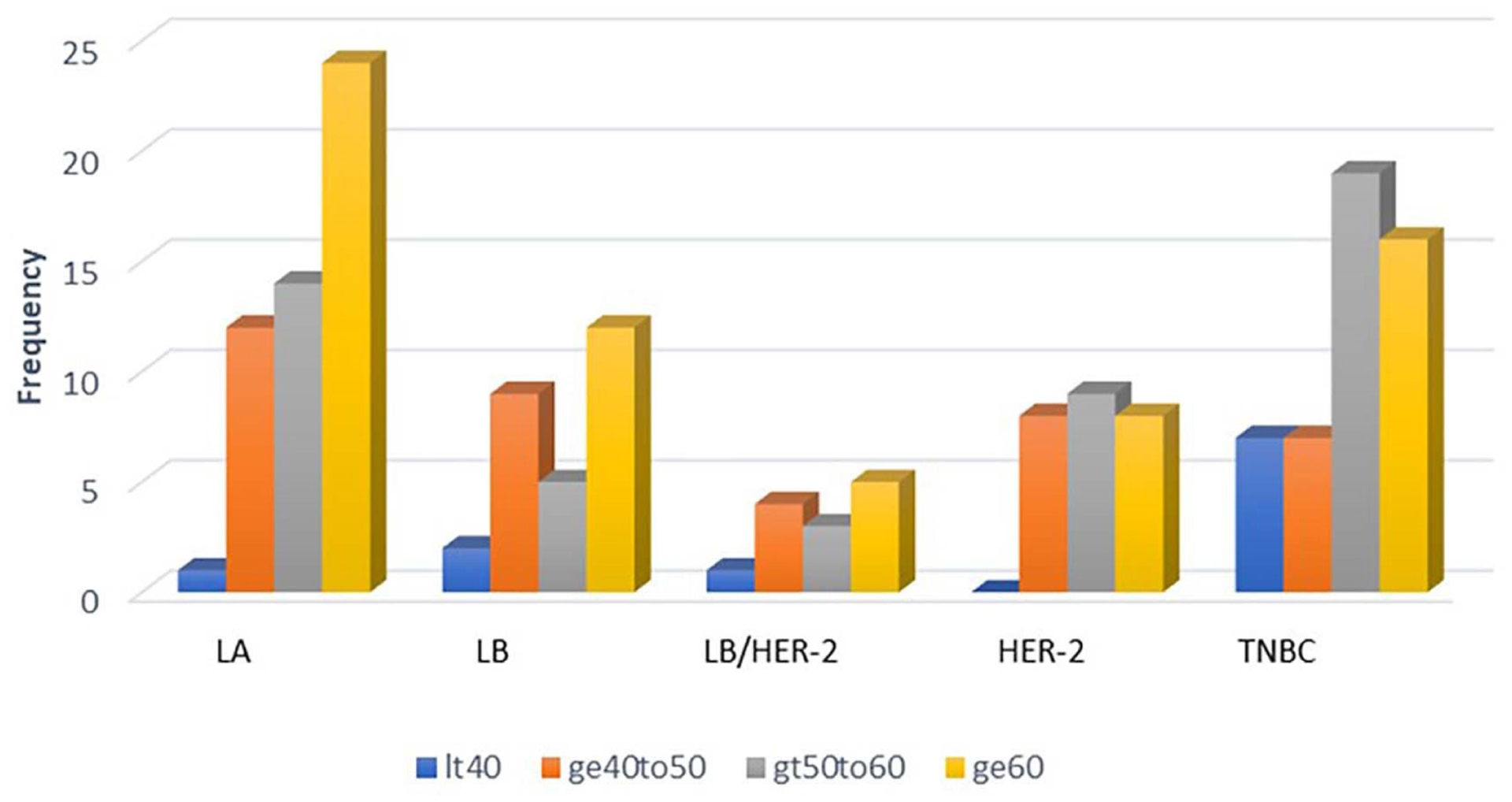
Distribution of patients according to age group within the breast cancer subtypes. LA: Luminal A; LB: Luminal B; LB/HER-2: Luminal B/ Human Epidermal growth factor Receptor 2; HER-2: Human Epidermal growth factor Receptor 2; TNBC: Triple Negative Breast Cancer. It40: age < 40; ge40to50: age ≥ 40 to 50; gt50to60: age > 50 to 60; ge60: age ≥60.

The age of patients was analyzed using one-factor ANOVA model and did not show any significant effect whether five (p-value= 0.576) or eight (p-value= 0.605) biomarkers were used, indicating that age did not have any influence on the classification of the subtypes. Thirty-one percent of the patients were less than 50 years old, 30% between 50 to 60 years and 39% older than 60 years (Figure 3).

### Overall survival and relapse-free survival

**Figure 4.**
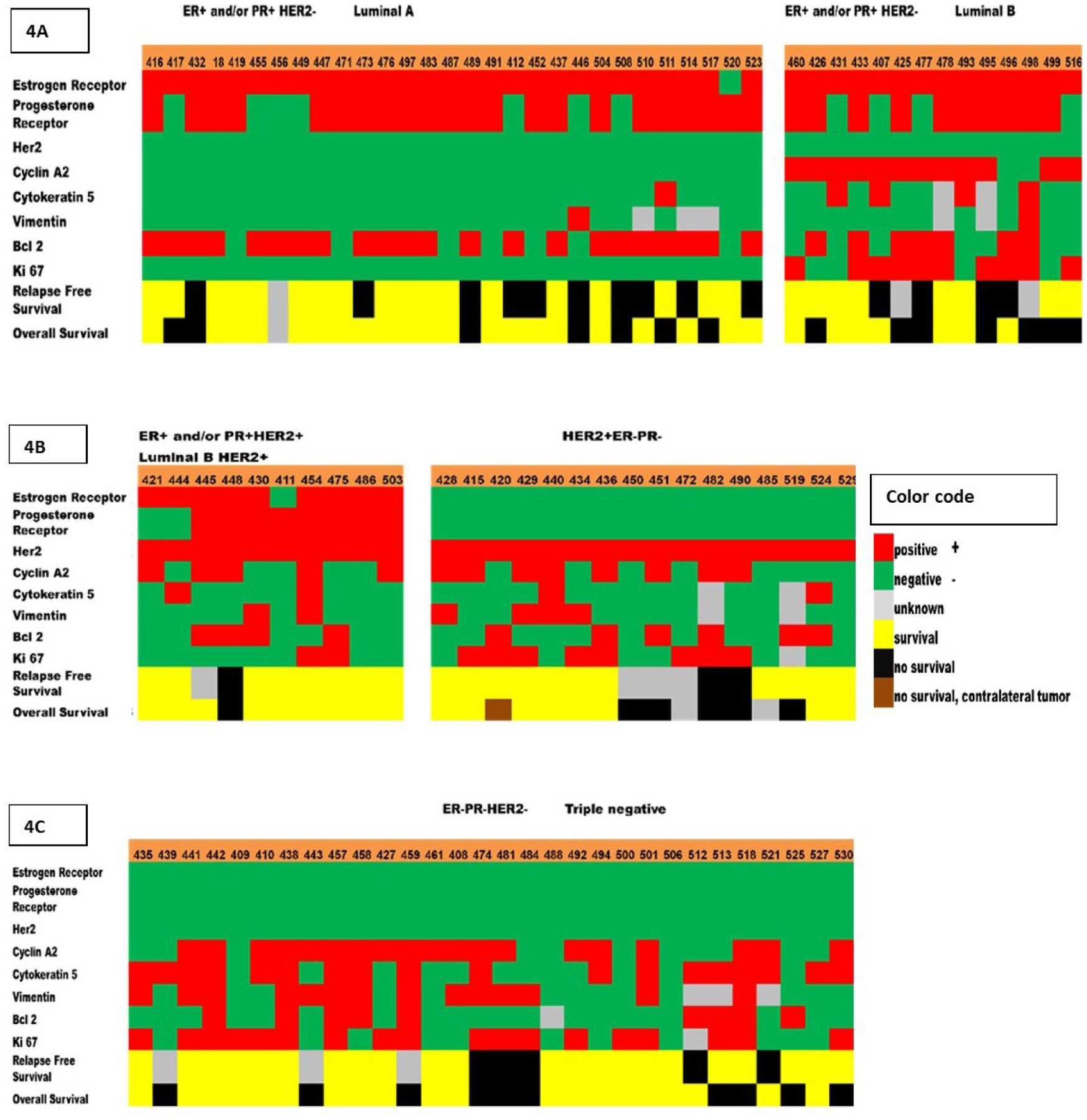
Heat map of the biomarkers expression that correspond to different breast tumor subtypes, recurrence, and survival. Five breast tumor subtypes were determined by immunostaining of the 8 protein biomarkers (ER, PR, HER-2, Cyclin A2, Ki-67, Bcl2, CK5, Vimentin). **(4a)** Luminal A (ER-positive and/or PR-positive, HER2-negative); Luminal B (ER-positive and/or PR-positive, HER2-negative, Cyclin A2-positive and/or Ki-67-positive). **(4b)** Luminal B HER2-positive (ER-positive and/or PR-positive, HER2-positive); HER2-positive (ER-negative, PR-negative, HER2-positive). **(4c)** Triple negative (ER-negative, PR-negative, HER2-negative). These subtypes are known to be associated with different relapse-free survival (RF) and overall survival (OS). Each column represents a sample, and each row represents the expression of one of the biomarkers. The expression is shown in different colors (color code).

Six-year overall survival was better for luminal A tumors (76%) than luminal B tumors (50%). Likewise, six-year relapse-free survival was better for luminal A tumors (76%) than luminal B tumors (29%) (Figure 4).

### Molecular classification of breast cancer subtypes

There was a significant overall agreement (95.8%) between the hierarchical clustering and multinomial logistic regression for the five different breast cancer subtypes (Table 4). The Kappa value of 0.95 and confidence interval (0.93, 0.99) indicate that the two tests gave comparable results. The p-value for the Chi-square test was 7.06e-^103^. The model with five biomarkers is a better model compared to the one with all biomarkers because they have the same overall percent correct clusters (95%) and Kappa value (0.932). The p-values for the Chi-square analysis are all less than 0.05, indicating that there is a high correlation between the predicted multinomial and hierarchical clustering and empirical classification. However, there was no significant difference in the distribution of samples within the five subtypes whether five (ER, PR, HER-2, Cyclin A2, Ki-67) or eight (ER, PR, HER-2, Cyclin A2, Ki-67, Bcl2, CK5, Vimentin) biomarkers were used for classification.

### Dendrogram and sequential hierarchical clustering

**Figure 5.**
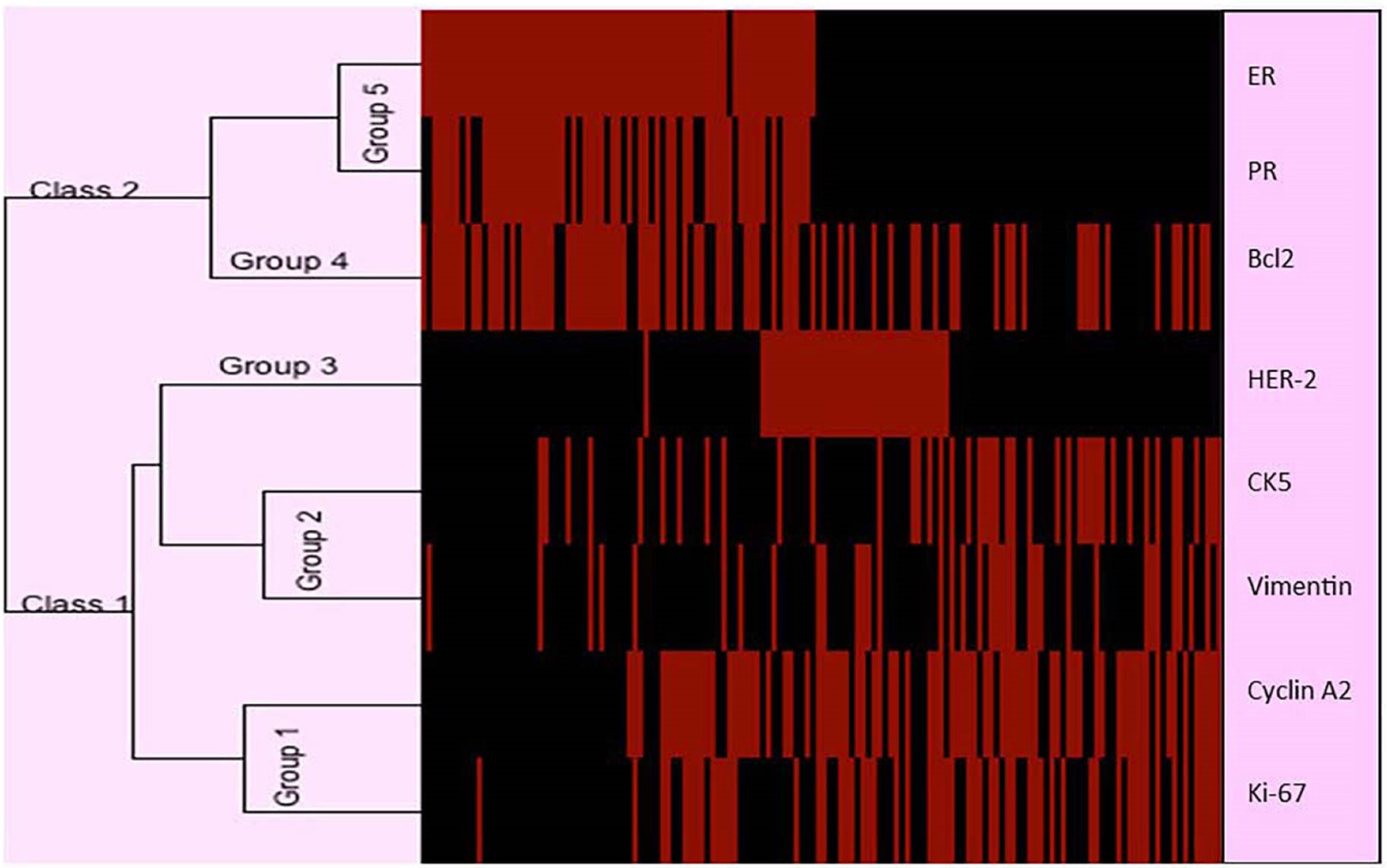
Dendrogram showing the expression profile of eight protein biomarkers based on immunohistochemistry. Five distinct protein expression profiles are observed: Group 1 consisting of Cyclin A2 and Ki-67; Group 2 consisting of CK5 and Vimentin; Group 3 and Group 4 consisting of HER-2 and Bcl2, respectively; Group 5 consisting of ER and PR. This classification is consistent with the functions of the proteins. Rows: Breast cancer tumors; Columns: Biomarkers proteins. In the dendrogram, the length of branch between two elements reflects their degree of relatedness. Red bars indicate a higher score in activation. Black bars indicate a lower score (less activation of the corresponding protein). ER: Estrogen Receptor; PR: Progesterone Receptor; HER-2: Human Epidermal growth factor Receptor 2; CK5: Cytokine 5; Bcl2: B-cell lymphoma 2.

The expression profile of the eight biomarkers in the tested breast cancer tissues is shown in Figure 5. Based on the dendrogram, five distinct protein expression profiles are observed: Group 1 consisting of Cyclin A2 and Ki-67; Group 2 consisting of CK5 and Vimentin; Group 3 and Group 4 consisting of HER-2 and Bcl2, respectively; Group 5 consisting of ER and PR. This classification is consistent with the function of the proteins.

**Figure 6.**
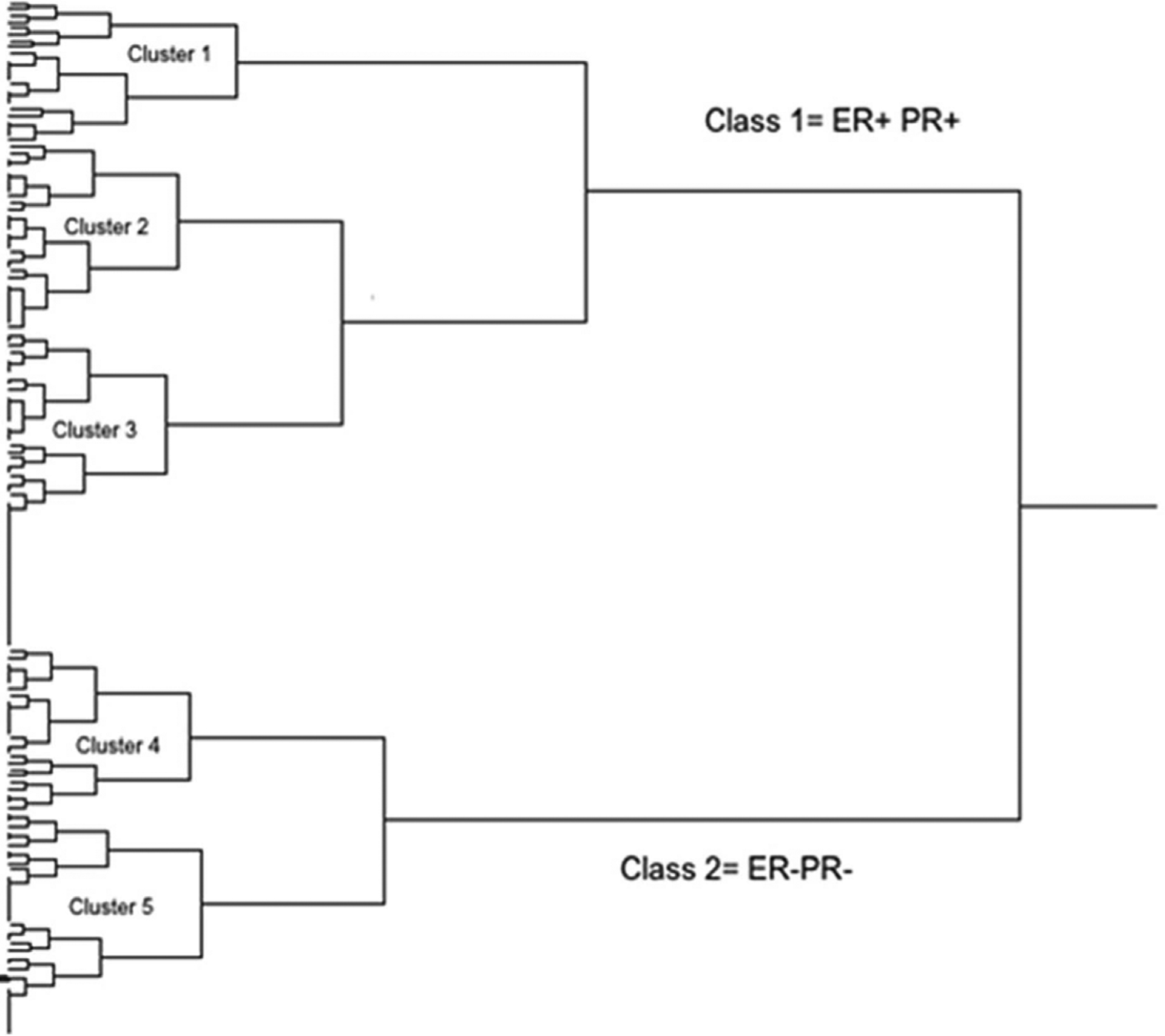
The sequential hierarchical clustering classifies the patients breast tumors into two Classes 1 and 2 based on ER and PR (using Ki-67 and Cyclin A2 to discriminate between luminal A and luminal B and rather only using HER-2 status). Hierarchical clustering using HER-2, Cyclin A2 and Ki-67 classifies Class 1 into clusters 1, 2 and 3; HER-2 by itself classifies Class 2 into clusters 4 and 5. The effect of each of the proteins within the five clusters is also shown in Figure 5. The significant effect of ER frequency is exhibited in clusters 1, 2 and 3. The effect of Cyclin A2 frequency is shown in cluster 1 while that of Ki-67 is shown in clusters 1 and 2. A significant effect of HER-2 frequency is shown for clusters 3 and 4. Cluster 1 - Luminal A: ER^+^ and/or PR^+^, HER-2^−^; Cluster 2 - Luminal B: ER^+^ and/or PR^+^, HER-2^−^ ; Cluster 3 - Luminal B HER-2: ER^+^ and/or PR^+^, HER-2^+^; Cluster 4 - HER-2 enriched: ER^−^, PR^−^, HER-2^+^; Cluster 5 - Triple-negative: ER^−^, PR^−^, HER-2^−^.

The sequential hierarchical clustering shown on the dendrogram in Figure 6 classifies the patient tumors into two Classes 1 and 2 based on ER and PR. Hierarchical clustering using HER-2, Cyclin A2 and Ki-67 classifies Class 1 into clusters 1, 2 and 3; HER-2 by itself classifies Class 2 into clusters 4 and 5. The effect of each of the proteins within the five clusters is also shown in Figure 5. The significant effect of ER frequency is exhibited in clusters 1, 2 and 3. The effect of Cyclin A2 frequency is shown in cluster 1 while that of Ki-67 is shown in clusters 1 and 2. A significant effect of HER-2 frequency is shown for clusters 3 and 4.

## Discussion

The existence of clinically distinct breast cancer subtypes has been established based on the protein level by using TMA technology [25,31,36,51]. Previous protein expression profiling studies have reported two to six different numbers of clusters. This variation may be due to the selected marker proteins, the number of breast cancer samples, and the type of used statistical method. Sorlie et al. [15,16] and Diallo-Dane Brock et al. [34] had identified five distinct subtypes of breast tumor that are associated with different clinical outcomes. The invasive breast carcinomas in our study were subclassified based on the clinicopathological surrogate definitions of subtypes as proposed by the 13^th^ St. Gallen International Breast Cancer Conference (2013) Expert Panel, based on immunohistochemical measurements of ER, PR, HER2, and Ki-67 with HER-2 in situ hybridization confirmation where appropriate [52]. Breast cancer subtypes were also classified [53] according to immunohistochemical profile as follows: luminal A [Hormone receptors (HR)-positive, HER-2 negative, Ki-67<14%], luminal B (HR-positive, HER-2 negative, Ki-67≥14%), luminal B/HER-2 (HR-positive and HER-2 positive), HER-2 enriched (HR-negative and HER-2 positive), and TNBC (HR- and HER-negative).

In the present study, we have identified five subtypes of breast tumors (Luminal A, Luminal B, Luminal B HER-2^+^, HER-2, and triple-negative) with distinct protein expression patterns among 166 African American women.

Most studies have applied unsupervised hierarchical clustering method to analyze the IHC scoring data [54–57]. In our study, we used both the supervised and unsupervised clustering methods and classified the tumor subtypes into ER^+^ and ER^−^ as well as the highly proliferative subgroups. ER and PR clustered together indicating similar function in breast tumor development. The association between ER and PR expression was statistically significant. This significant association between ER and PR in the TMA has also been reported in Danish patients by Henriksen et al. [58] using semi-quantitative IHC analyses. Cyclin A2 and Ki-67 clustered together as proliferation markers. This proliferation cluster is remarkably like that identified by Callogy [47], Korsching [59], and Ameh-Mensah [60] in British, German, and Ghanaian patients; respectively.

Our results have identified five different subtypes with a greater proportion of the patients (60%) belonging to luminal A and triple-negative subtypes. Li et al. [61] found a highest percentage of luminal A (35.6%), followed by luminal B (22.5%), HER2-positive luminal B (13.1%), triple negative (15.2%), and non-luminal HER2-positive (13.7%) tumors from Chinese patients. Van Leered et al. [62] found a significant higher fraction of inflammatory breast cancer in Norwegian patients belongs to the HER-2 and basal-like subtype. Cheang et al. [40] also identified 59% as Luminal A, 33% as Luminal B tumors, and 9% as Luminal B HER-2^+^ tumors from British patients. Zhou [63] found out 186 Swedish patients of those classified as ductal carcinoma in situ (DCIS) were Luminal A (48.8%), 33 Luminal B/HER-2^−^ (8.7%), 74 Luminal B/HER-2+ (17.4%), 61 HER-2_+_/ER^−^ (16.0%) and 27 triple negative (7.1%). Similarly, in our study of 94% hormone receptor positive tumors, 55% were luminal A, 30% were luminal B and 15% were luminal B HER-2^+^. In Cheang study, only 21% of the Luminal B carcinomas were luminal B HER-2^+^ whereas in our study 32% of luminal B tumors are luminal B HER-2^+^. Gene expression microarray studies demonstrated that about 30% of the luminal B tumors are ER^+^ and/or PR^+^/HER-2^+^; whereas 70% of the luminal B carcinomas have the same ER^+^ and/or PR^+^, HER-2^−^ profile as luminal A tumors in African American [3]. It is worth noting that 68% of the carcinomas in the luminal B subtype in our study had the same profile as luminal A tumors. Since both luminal A and luminal B tumors are ER^+^ and/or PR^+^/ HER-2^−^, differentiating between luminal A and luminal B subtypes is particularly important in terms of treatment and survival because luminal A patients are known to have a better survival than luminal B tumors and all other subtypes [40,64]. African American patients presented with advanced stage tumors and higher histologic and nuclear grade than Caucasian patients; and less ER positivity but significantly higher Ki-67 and p53 expression than Caucasian patients with all stages of disease [11].

Previous studies of African Americans at Howard University Hospital did not differentiate the luminal A tumors from the more aggressive luminal B tumors [65,66]. Results from our present showed that Cyclin A2 and Ki-67 clearly distinguished luminal A from luminal B. Of the 166 samples that stained with either Cyclin A2 or Ki-67 antibody, 49% were positive for Cyclin A2; 39.2 % positive for Ki-67 (>41%); and only 26% positive for both Cyclin A2 and Ki-67. Therefore, immunostaining of both Cyclin A2 and Ki-67 are recommended for immuno-histological identification of tumors with poor prognosis, which may aid physicians in treatment decisions. These two proliferation markers were common in the poor prognosis ER^+^/PR^+^/HER^−^ tumors. Cheang et al. [40] and Hu et al. [67] used Ki-67 to distinguish luminal A from luminal B tumors with the same ER^+^/PR^+^/HER-2^−^ profile. Differentiating between luminal A and luminal B subtypes is clinically important because luminal B tumors have a worse prognosis and often show a better response to treatment chemotherapy than luminal A [40,64]. Luminal B tumors are hormone-insensitive and chemo-insensitive with poor survival [68,69]. In our study, luminal B tumors also had worse overall survival (55%) than luminal A tumors (71%). Likewise, six-year relapse-free survival was better for luminal A tumors (76%) than luminal B tumors (29%). Our study shows that Bcl2 was higher in luminal A than luminal B tumors. Eom et al. [70] reported that the prognostic role of Bcl2 expression in breast cancer is subtype-specific; and Bcl2 expression differs according to the molecular subtype and is a good prognostic marker for only luminal A breast cancer in Korean patients. Previous studies have demonstrated that proliferation markers are associated with high histological grade and poor prognosis. In the Oncotype Dx test, proliferation markers Ki-67 and Cyclin B1, are major factors in calculating recurrence in hormone positive-node negative tumors [71]. Poikonen et al. [72] have observed that Cyclin A2 protein overexpression is a better marker for poor prognosis (risk ratio, first relapse time, survival) than Ki-67, histological grade, or mitotic count in breast cancer patients. Aaltonen et al. [39] have observed that Cyclin A2 protein was significantly detected in ER^−^/PR^−^ high grade breast tumors and connected with worse metastasis-free survival in Finish patients. Soliman and colleagues [73] reported that Luminal A patients with Ki-67 less than 15% displayed better overall survival than luminal B with Ki-67 higher than 15%; and Ki-67 may be considered a valuable biomarker in breast cancer Egyptian patients. As for Ki-67 expression in breast cancer cells, the score increases with increase of tumor size, grade, premenopausal. Ki-67 expression in ER and PR receptor positive tumors showed lower values than ER and PR negative tumors, while higher Ki-67 expression was more frequently associated with HER2-positive (74). Wajid et all reported that expression of ER, PR, and HER-2 resembled that of Western countries with no differences between urban and rural centers of Egypt and most Egyptian cases were classified as Luminal A (44%), which offers the best prognosis of all the subtypes. Elevated Cyclin A2 stimulates oncogenesis by promoting G1/S and G2/M transitions in the cell cycle [75,76]. HER-2 enriched subtype in our study was only 15% of the total 166 patient samples. This subtype is hormone receptor weak/negative, Cyclin A2 positive and shows a high expression of basal type proteins CK5 and Vimentin. In our study, eighty percent (20/25) of the HER-2^+^/ER^−^/PR^−^ tumors were positive for Cyclin A2 and/or Ki-67. According to Morris et al. [11] approximately 30% of breast cancers have an amplification of the HER-2/neu gene or overexpression of its protein product. Overexpression of this receptor in breast cancer is associated with increased disease recurrence and worse prognosis.

**Figure 7.**
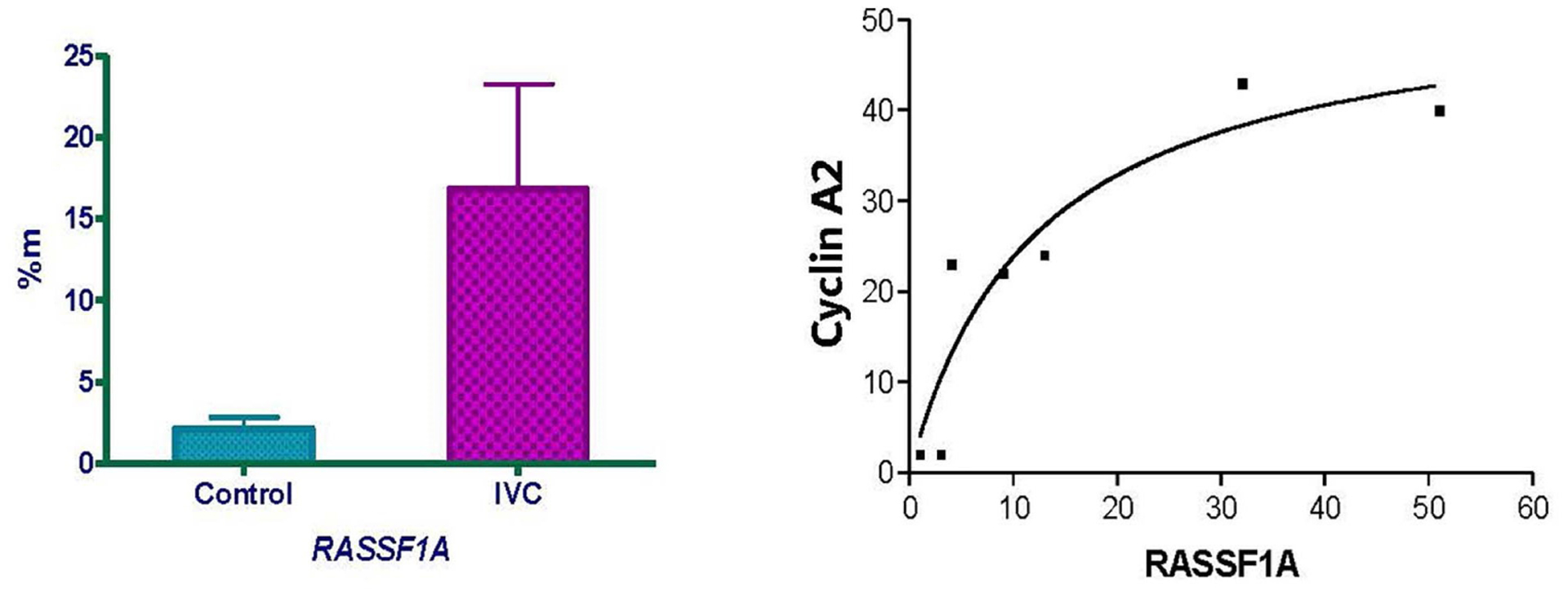
Direct relationship between RASSF1A methylation and Cyclin A2 protein staining in normal and ER-negative/PR-negative/HER2-negative samples. Methylation of the CpG islands in the RASSF1A promoter is expected to decrease RASSF1A transcription and thereby increase Cyclin A2 transcription and protein levels. We observed significantly (p=0.039) increased levels of RASSF1A methylation (%m) in triple negative invasive ductal carcinoma compared to mammoplasty samples in African American (Figure 7, left). Increased Cyclin A2 protein staining correlated with increased RASSF1A methylation (Figure 7, right). These data suggest that methylation of RASSF1A is one mechanism by which Cyclin A2 protein levels are elevated in African American breast cancers.

According to Pratt et al. [68] many triple-negative tumors were either basal-like (39% to 54%) or Claudin-low (25% to 39%). DNA microarray study by Prat & Peron [69] showed that triple-negative tumors consist of basal (50%), claudin-low (30%), HER-2^+^ (9%), luminal B (6%) and luminal A (5%) subtypes. The triple-negative group as defined by immunohistochemical staining consists for approximately 80% of basal-like breast cancers as defined by gene expression profiling [77]. Our triple-negative tumors have many of the characteristics of basal tumors: invasive ductal carcinoma, grade 3 tumors, CK5+, Vimentin+, highly proliferative markers (increased Ki-67^+^ and Cyclin A2^+^). Eighty-four percent (41/49) of the triple-negative (ER^−^/PR^−^/HER-2^−^) tumors were also positive for the proliferation markers Cyclin A2 and/or Ki-67. The basal markers CK5 and vimentin were also common in the triple-negative tumors, as expected. Seventy-five percent of the HER-2^+^/ER^−^/PR^−^ tumors were positive for Cyclin A2 and/or Ki-67. The RASSF1A protein has been reported to inhibit the transcription of Cyclin A2 [78]. Methylation of the CpG islands in the RASSF1A promoter is expected to decrease RASSF1A transcription and thereby increase Cyclin A2 transcription and protein levels. We observed significantly increased levels of RASSF1A methylation (%m) with African American triple-negative breast cancer tumors compared to mammoplasty samples (Figure 7). Increased Cyclin A2 protein staining correlated with increased RASSF1A methylation (Figure 7). These data suggest that methylation of RASSF1A is one mechanism by which Cyclin A2 protein levels are elevated in African American breast tumors.

Earlier studies at Howard University Hospital demonstrated that African American triple-negative tumors and HER-2^+^/ER^−^/PR^−^ carcinomas had the poorest breast cancer-specific survival [65]. Furthermore, triple-negative tumors in African American women at Howard University Hospital were significantly associated with a higher incidence of distant metastases [66]. Therefore, it appears that the proliferation proteins, Cyclin A2 and Ki-67, are associated with poor survival among African American breast cancer patients.

## Conclusion

We identified five different subtypes with a greater proportion of the patients (60%) belongs to luminal A and triple-negative subtypes. Furthermore, our findings have shown that both Cyclin A2 and Ki-67 proliferation markers can be used for immune-histological identification of breast tumors with poor prognosis. This will aid physicians in making decisions on the treatment of the tumors. Previous studies have demonstrated that proliferation markers are associated with high histological grade and poor prognosis. Our findings are consistent with the previously reported data by other investigators. Therefore, discovery of molecular markers and subtypes that are most prevalent in African Americans could lead to a better understanding of the factors contributing to higher mortality in this group and to better treatment.

## Contribution to the Field Statement

It is well established that racial differences can influence breast cancer incidence and mortality. Diagnosed invasive breast carcinomas in African American patients are more aggressive compared with those in Caucasian patients and diagnosed at later stages of the disease with higher grade tumors. African American patients with breast carcinomas are more likely than Caucasian patients to present with tumors that are of a later stage and higher grade, with higher Ki-67 expression and more ER negativity [79].

Earlier studies at Howard University Hospital (HUH) demonstrated that African American triple-negative tumors and HER-2^+^/ER^−^/PR^−^ carcinomas had the poorest breast cancer-specific survival. Furthermore, triple-negative tumors in African American women at HUH were significantly associated with a higher incidence of distant metastases. However, these studies did not differentiate the luminal A tumors from the more aggressive luminal B tumors. Results from our study showed that Cyclin A2 and Ki-67 clearly distinguished luminal A from luminal B. Also, our triple-negative tumors have many of the characteristics of basal tumors: invasive ductal carcinoma, grade 3 tumors, CK5+, Vimentin+, highly proliferative markers (increased Ki-67^+^ and Cyclin A2^+^). Eighty-four percent of the triple-negative tumors were also positive for the proliferation markers Cyclin A2 and/or Ki-67. Furthermore, it appears that the proliferation proteins, Cyclin A2 and Ki-67, are associated with poor survival among African American breast cancer patients. Personalized medicine to decrease the adverse effects of chemotherapy is not only requires clinical, but also molecular characterization of tumors, which allows the use of more effective drugs for each patient.

Therefore, both Cyclin A2 and Ki-67 are recommended for immuno-histological identification of tumors with poor prognosis and thus aid physicians in treatment decisions. These data may heighten the need to employ these techs to increase detection rates in this high-risk population.

## Limitations

The number of protein biomarkers and patients’ samples in the used TMAs for subclassifying tumors into clinically and biologically relevant subgroups were limited. For the study, based on the available resources, we choose to follow the expert panel recommendations and the prognostic significance of Cyclin A2 and Ki-64 in breast cancer mainly in African American women which needs evaluation in larger studies. PAM50 (Prediction Analysis of Microarray 50) molecular testing is not performed on our study population, and we will consider this in our future study to predict the chance of metastasis for some ER-positive, HER2-negative breast cancers. Also, inclusion of CCNA2 immunohistochemistry for the identification of the luminal B subtype.

## Figures and Tables

**Table 1.** List of the investigated markers and characteristics of the corresponding dilution of the specific anti-human antibody used in immunohistochemistry study. Staining characteristic: expression location in the cell (nuclear, membranous, cytoplasmic). ER: Estrogen; PR: Progesterone; HER-2: Human epidermal growth factor receptor 2; Bcl2: B-cell lymphoma 2; CK5: Cytokine 5.

Markers	Antibodies	Clone	Dilution	Source	Expression
ER	Rabbit monoclonal	SP-1	1:100	Dako	Nuclear
PR	Mouse monoclonal	PgR636	1:500	Dako	Nuclear
HER-2	Rabbit polyclonal	e-erb-2	1:200	Dako	Membranous
Cyclin A2	Mouse monoclonal	6E6	1:50	Novacastra	Nuclear
Bcl2	Mouse monoclonal	124	1:100	Dako	Cytoplasmic
CK5	Mouse monoclonal	XM26	1:150	Novacastra	Cytoplasmic
Ki-67	Mouse monoclonal	MIB-1	1:100	Dako	Nuclear
Vimentin	Mouse monoclonal	V9	1:100	Dako	Cytoplasmic

**Table 2.** Descriptive statistical analysis showing the number of patients (N) with positive and negative expressions for the tested 8 biomarkers. The mean age differences with standard deviation (SD) and 95% confidence intervals (CI) are only included since age did not show any significant effect on the expression of the 8 biomarkers.

Biomarkers expression	N	Mean (age)	SD (age)	Minimum (age)	Maximum (age)	95% CI Mean (age)
**ER**						
Negative	76	55.62	12.77	32	96	[52.70,58.53]
Positive	90	59.2	14.09	29	96	[56.25,62.15]
**PR**						
Negative	100	56.68	12.89	32	96	[54.12,59.23]
Positive	66	58.89	14.56	29	86	[55.31,62.47
**HER-2**						
Negative	126	57.81	13.91	29	86	[55.36,60.26]
Positive	40	56.78	12.64	32	96	[52.73,60.81]
**Cyclin A2**						
Negative	85	58.29	13.85	29	96	[55.28,61.30]
Positive	81	56.79	13.225	32	86	[53.87,59.71]
**Bcl2**						
Negative	74	55.73	12.667	32	85	[52.79,58.66]
Positive	92	59.03	14.17	29	96	[56.09,61.97]
**CK5**						
Negative	121	58.23	12.953	34	86	[55.89,60.56]
Positive	45	55.76	15.151	29	96	[51.20,60.31]
**Vimentin**						
Negative	129	58.65	13.369	29	96	[56.32,60.98]
Positive	37	53.76	13.813	32	85	[49.15,58.36]
**Ki-67**						
Negative	101	58.08	13.611	29	96	[55.39,60.77]
Positive	65	56.75	13.602	33	86	[53.38,60.12]

**Table 3.** Distribution of the166 patient samples among clusters based on hierarchical clustering and multinomial regression.

			Multinomial logistic regression	
Cluster	Empirical	Hierarchical	8 markers	5 markers
Subtype	number (%)	number (%)	number (%)	number (%)
Luminal A	51 (30.7)	46 (27.7)	51 (30.7)	49 (29.5)
Luminal B	27 (16.3)	34 (20.5)	28 (16.9)	28 (16.9)
Luminal B (HER-2^+^)	16 (9.6)	13 (7.8)	13 (7.8)	15 (9.0)
HER-2	23 (13.9)	24 (14.5)	25 (15.1)	25 (15.1)
Triple-negative	49 (29.5)	49 (29.5)	49 (29.5)	49 (29.5)

**Table 4.** Test of agreement between the hierarchical clustering and multinomial regression for the different subtypes using five markers. The percent agreement between using five and eight biomarkers was 95.8%.

Subtypes	Empirical Vs hierarchical	Empirical Vs multinomial	Hierarchical Vs multinomial
Luminal A	0.91	0.93	0.97
Luminal B	0.82	0.86	0.90
Luminal B HER-2^+^	0.9	0.95	0.86
HER	0.97	0.97	0.98
Triple-negative	1.0	1.0	1.0
Kappa	0.91	0.93	0.95
Overall Percent correct	93%	95%	95.8%
CI of Overall Percent correct 0.95 level	[0.89,0.98]	[0.91,0.99]	[0.93,0.99]
Chi-square (p-value)	1.04 e-^84^	1.73 e-^87^	7.06 e-^103^

## References

[1] Siegel RL, Miller KD, Fuchs HE, Jemal A. Cancer statistics, 2022. CA: A Cancer Journal for Clinicians. 2022 Jan;72(1):7–33. 35020204 10.3322/caac.21708

[2] https://www.cancer.org/content/dam/CRC/PDF/Public/8577.00.pdf

[3] Stringer-Reasor EM, Elkhanany A, Khoury K, Simon MA, Newman LA. Disparities in breast cancer associated with African American identity. American Society of Clinical Oncology Educational Book. 2021 May 27;41:e29–46. 34161138 10.1200/EDBK_319929

[4] Howlader N, Noone AM, Krapcho M, Miller D, Brest A, Yu M, Ruhl J, Tatalovich Z, Mariotto A, Lewis DR, Chen HS. SEER cancer statistics review, 1975–2016, National Cancer Institute. Bethesda, MD. 2019 Apr;2020:1–0.

[5] Newman LA. Breast cancer in minority women. Advanced Therapy of Breast Disease, 2nd ed. Hamilton: BC Decker Inc. 2004:713–26.

[6] Newman LA. Breast cancer in African-American women. Oncologist. 2005 Jan;10(1):1–14. 10.1634/theoncologist.10-1-1 15632248

[7] Hirko KA, Rocque G, Reasor E, Taye A, Daly A, Cutress RI, Copson ER, Lee DW, Lee KH, Im SA, Park YH. The impact of race and ethnicity in breast cancer—disparities and implications for precision oncology. BMC Medicine. 2022 Dec;20(1):72. 35151316 10.1186/s12916-022-02260-0 PMC8841090

[8] Joslyn SA, West MM. Racial differences in breast carcinoma survival. Cancer. 2000 Jan 1;88(1):114–23. 10618613 10.1002/(sici)1097-0142(20000101)88:1<114::aid-cncr16>3.0.co;2-j

[9] Copson E, Maishman T, Gerty S, Eccles B, Stanton L, Cutress RI, Ethnicity and outcome of young breast cancer patients in the United Kingdom: the POSH study. British Journal of Cancer. 2014 Jan;110(1):230–41. 24149174 10.1038/bjc.2013.650 PMC3887284

[10] Jemal A, Robbins AS, Lin CC, Flanders WD, DeSantis CE, Ward EM, Factors that contributed to black-white disparities in survival among nonelderly women with breast cancer between 2004 and 2013. Journal of Clinical Oncology. 2018 Jan 1;36(1):14–24. 29035645 10.1200/JCO.2017.73.7932

[11] Morris GJ, Naidu S, Topham AK, Guiles F, Xu Y, McCue P, Differences in breast carcinoma characteristics in newly diagnosed African–American and Caucasian patients: A single-institution compilation compared with the National Cancer Institute’s Surveillance, Epidemiology, and end results database. Cancer: Interdisciplinary International Journal of the American Cancer Society. 2007 Aug 15;110(4):876–84. 10.1002/cncr.22836 17620276

[12] Hagerty RG, Butow PN, Ellis PM, Lobb EA, Pendlebury SC, Leighl N, Communicating with realism and hope: incurable cancer patients’ views on the disclosure of prognosis. Journal of Clinical Oncology. 2005 Feb 20;23(6):1278–88. 15718326 10.1200/JCO.2005.11.138

[13] Zavala VA, Bracci PM, Carethers JM, Carvajal-Carmona L, Coggins NB, Cruz-Correa MR, Cancer health disparities in racial/ethnic minorities in the United States. British Journal of Cancer. 2021 Jan 19;124(2):315–32. 32901135 10.1038/s41416-020-01038-6 PMC7852513

[14] Allinen M, Beroukhim R, Cai L, Brennan C, Lahti-Domenici J, Huang H, Molecular characterization of the tumor microenvironment in breast cancer. Cancer Cell. 2004 Jul 1;6(1):17–32. 15261139 10.1016/j.ccr.2004.06.010

[15] Sørlie T, Perou CM, Tibshirani R, Aas T, Geisler S, Johnsen H, Gene expression patterns of breast carcinomas distinguish tumor subclasses with clinical implications. Proceedings of the National Academy of Sciences. 2001 Sep 11;98(19):10869–74. 10.1073/pnas.191367098 PMC58566 11553815

[16] Sørlie T, Tibshirani R, Parker J, Hastie T, Marron JS, Nobel A, Repeated observation of breast tumor subtypes in independent gene expression data sets. Proceedings of the National Academy of Sciences. 2003 Jul 8;100(14):8418–23. 10.1073/pnas.0932692100 PMC166244 12829800

[17] Crowe JP Jr, Patrick RJ, Rybicki LA, Grundfest-Broniatowski S, Kim JA, Lee KB. Race is a fundamental prognostic indicator for 2325 northeastern Ohio women with infiltrating breast cancer. The Breast Journal. 2005 Mar;11(2):124–8. 15730458 10.1111/j.1075-122X.2005.21564.x

[18] Winer EP, Carey LA, Dowsett M, Tripathy D, Perry MC. Beyond anatomic staging: are we ready to take the leap to molecular classification. In2005 ASCO Annual Meeting 2005 (pp. 46–59).

[19] Allison KH. Prognostic and predictive parameters in breast pathology: a pathologist’s primer. Modern Pathology. 2021 Jan 1;34:94–106. 33154551 10.1038/s41379-020-00704-7

[20] Carey LA, Perou CM, Livasy CA, Dressler LG, Cowan D, Conway K, Race, breast cancer subtypes, and survival in the Carolina Breast Cancer Study. Jama. 2006 Jun 7;295(21):2492–502. 16757721 10.1001/jama.295.21.2492

[21] Zaha DC, Lazar E, Lazureanu C. Clinicopathologic features and five years survival analysis in molecular subtypes of breast cancer. Rom J Morphol Embryol. 2010 Jan 1;51(1):85–9. 20191125

[22] Prat A, Pineda E, Adamo B, Galván P, Fernández A, Gaba L, Clinical implications of the intrinsic molecular subtypes of breast cancer. The Breast. 2015 Nov 1;24:S26–35. 26253814 10.1016/j.breast.2015.07.008

[23] Parker SL, Davis KJ, Wingo PA, Ries LA, Heath CW Jr. Cancer statistics by race and ethnicity. CA: A Cancer Journal for Clinicians. 1998 Jan 1;48(1):31–48. 9449932 10.3322/canjclin.48.1.31

[24] Henson DE, Chu KC, Levine PH. Histologic grade, stage, and survival in breast carcinoma: comparison of African American and Caucasian women. Cancer. 2003 Sep 1;98(5):908–17. 12942556 10.1002/cncr.11558

[25] Abd El-Rehim DM, Ball G, Pinder SE, Rakha E, Paish C, Robertson JF, High-throughput protein expression analysis using tissue microarray technology of a large well-characterised series identifies biologically distinct classes of breast cancer confirming recent cDNA expression analyses. International journal of cancer. 2005 Sep 1;116(3):340–50. 15818618 10.1002/ijc.21004

[26] Hurd TC, Chapman IR, Kelly M, Wall B, Rodgers T, Womack SD. Factors affecting breast cancer screening practices among African American women in Western New York. Breast Cancer Research and Treatment. 2005 Jan 1;94(Suppl 1):S157.

[27] Maloney N, Koch M, Elkins D. Racial differences in breast cancer of lower socio-economic status women. Breast Cancer Res Treat. 2004.

[28] Newman LA, Griffith KA, Jatoi I, Simon MS, Crowe JP, Colditz GA. Meta-analysis of survival in African American and white American patients with breast cancer: ethnicity compared with socioeconomic status. J Clin Oncol. 2006 Mar 20;24(9):1342–9. 16549828 10.1200/JCO.2005.03.3472

[29] Li H, Brewer G, Ongo G, Normandeau F, Omeroglu A, Juncker D. Immunohistochemistry microarrays. Analytical Chemistry. 2017 Sep 5;89(17):8620–5. 28763195 10.1021/acs.analchem.7b00807

[30] Sun X, Shan Y, Li Q, Chollet-Hinton L, Kirk EL, Gierach GL, Intra-individual gene expression variability of histologically normal breast tissue. Scientific Reports. 2018 Jun 14;8(1):9137. 29904148 10.1038/s41598-018-27505-y PMC6002361

[31] Makretsov NA, Huntsman DG, Nielsen TO, Yorida E, Peacock M, Cheang MC, Hierarchical clustering analysis of tissue microarray immunostaining data identifies prognostically significant groups of breast carcinoma. Clinical Cancer Research. 2004 Sep 15;10(18):6143–51. 15448001 10.1158/1078-0432.CCR-04-0429

[32] Jacquemier J, Ginestier C, Rougemont J, Bardou VJ, Charafe-Jauffret E, Geneix J, Protein expression profiling identifies subclasses of breast cancer and predicts prognosis. Cancer Research. 2005 Feb 1;65(3):767–79. 15705873

[33] Dolled-Filhart M, Rydén L, Cregger M, Jirström K, Harigopal M, Camp RL, Classification of breast cancer using genetic algorithms and tissue microarrays. Clinical Cancer Research. 2006 Nov 1;12(21):6459–68. 17085660 10.1158/1078-0432.CCR-06-1383

[34] Diallo-Danebrock R, Ting E, Gluz O, Herr A, Mohrmann S, Geddert H, Protein expression profiling in high-risk breast cancer patients treated with high-dose or conventional dose–dense chemotherapy. Clinical Cancer Research. 2007 Jan 15;13(2):488–97. 17255270 10.1158/1078-0432.CCR-06-1842

[35] Allred DC, Bustamante MA, Daniel CO, Gaskill HV, Cruz AB. Immunocytochemical analysis of estrogen receptors in human breast carcinomas: evaluation of 130 cases and review of the literature regarding concordance with biochemical assay and clinical relevance. Archives of Surgery. 1990 Jan 1;125(1):107–13. 1688490 10.1001/archsurg.1990.01410130113018

[36] McKenna SJ, Amaral T, Akbar S, Jordan L, Thompson A. Immunohistochemical analysis of breast tissue microarray images using contextual classifiers. Journal of Pathology Informatics. 2013 Jan 1;4(2):13. 23766935 10.4103/2153-3539.109871 PMC3678746

[37] Detre S, Jotti GS, Dowsett M. A” quickscore” method for immunohistochemical semiquantitation: validation for oestrogen receptor in breast carcinomas. Journal of Clinical Pathology. 1995 Sep 1;48(9):876–8. 7490328 10.1136/jcp.48.9.876 PMC502883

[38] Hewitt SM. Tissue microarrays as a tool in the discovery and validation of predictive biomarkers. Molecular Profiling: Methods and Protocols. 2012:201–14. 10.1007/978-1-60327-216-2_13 PMC7457565 22081347

[39] Aaltonen K, Ahlin C, Amini RM, Salonen L, Fjällskog ML, Heikkilä P, Reliability of cyclin A assessment on tissue microarrays in breast cancer compared to conventional histological slides. British Journal of Cancer. 2006 Jun;94(11):1697–702. 16670718 10.1038/sj.bjc.6603147 PMC2361315

[40] Cheang MC, Chia SK, Voduc D, Gao D, Leung S, Snider J, Ki67 index, HER2 status, and prognosis of patients with luminal B breast cancer. JNCI: Journal of the National Cancer Institute. 2009 May 20;101(10):736–50. 19436038 10.1093/jnci/djp082 PMC2684553

[41] Veronese SM, Maisano C, Scibilia J. Comparative prognostic value of Ki-67 and MIB-1 proliferation indices in breast cancer. Anticancer research. 1995 Nov 1;15(6B):2717–22. 8669852

[42] 31 IBM Corp. Released 2011. IBM SPSS Statistics for Windows, Version 20.0 Armonk, NY: IBM Corp.

[43] De Hoon MJ, Imoto S, Nolan J, Miyano S. Open source clustering software. Bioinformatics. 2004 Jun 12;20(9):1453–4. 14871861 10.1093/bioinformatics/bth078

[44] Hintze J (2013). NCSS 9. Spreadsheet or database NCSS, LLC Kaysville, Utah, USA. www.ncss.com.

[45] Cox DR. Regression models and life-tables. Journal of the Royal Statistical Society: Series B (Methodological). 1972 Jan;34(2):187–202.

[46] Cox DR. Regression models and life table. J R Stat Soc B. 1972;34:187–220.

[47] Callagy G, Cattaneo E, Daigo Y, Happerfield L, Bobrow LG, Pharoah PD, Molecular classification of breast carcinomas using tissue microarrays. Diagnostic Molecular Pathology. 2003 Mar 1;12(1):27–34. 12605033 10.1097/00019606-200303000-00004

[48] Abd El-Rehim DM, Ball G, Pinder SE, Rakha E, Paish C, Robertson JF, High-throughput protein expression analysis using tissue microarray technology of a large well-characterised series identifies biologically distinct classes of breast cancer confirming recent cDNA expression analyses. International Journal of Cancer. 2005 Sep 1;116(3):340–50. 15818618 10.1002/ijc.21004

[49] Cicchetti DV, Feinstein AR. High agreement but low kappa: II. Resolving the paradoxes. Journal of Clinical Epidemiology. 1990 Jan 1;43(6):551–8. 2189948 10.1016/0895-4356(90)90159-m

[50] Ryan J, Pereira RH. What are we missing when we measure accessibility? Comparing calculated and self-reported accounts among older people. Journal of Transport Geography. 2021 May 1;93:103086.

[51] Sheen-Chen SM, Huang CY, Liu YY, Huang CC, Tang RP. Cortactin in breast cancer: analysis with tissue microarray. Anticancer Research. 2011 Jan 1;31(1):293–7. 21273613

[52] Goldhirsch A, Winer EP, Coates AS, Gelber RD, Piccart-Gebhart M, Thürlimann B, Senn HJ, Albain KS, André F, Bergh J, Bonnefoi H. Personalizing the treatment of women with early breast cancer: highlights of the St Gallen International Expert Consensus on the Primary Therapy of Early Breast Cancer 2013. Annals of Oncology. 2013 Sep 1;24(9):2206–23. 23917950 10.1093/annonc/mdt303 PMC3755334

[53] Ignatov A, Eggemann H, Burger E, Ignatov T. Patterns of breast cancer relapse in accordance to biological subtype. Journal of cancer Research and Clinical Oncology. 2018 Jul;144:1347–55. 29675790 10.1007/s00432-018-2644-2 PMC11813410

[54] Kruger DT, Beelen KJ, Opdam M, Sanders J, van der Noort V, Boven E, Hierarchical clustering of activated proteins in the PI3K and MAPK pathways in ER-positive, HER2-negative breast cancer with potential therapeutic consequences. British Journal of Cancer. 2018 Oct 2;119(7):832–9. 30287915 10.1038/s41416-018-0221-8 PMC6189147

[55] Kruger DT, Opdam M, Sanders J, van der Noort V, Boven E, Linn SC. Hierarchical clustering of PI3K and MAPK pathway proteins in breast cancer intrinsic subtypes. Apmis. 2020 Apr;128(4):298–307. 31976581 10.1111/apm.13026 PMC7317370

[56] Kruger DT, Opdam M, van der Noort V, Sanders J, Nieuwenhuis M, de Valk B, PI3K pathway protein analyses in metastatic breast cancer patients receiving standard everolimus and exemestane. Journal of Cancer Research and Clinical Oncology. 2020 Nov;146:3013–23. 32566979 10.1007/s00432-020-03291-x PMC7519923

[57] Ferro S, Bottigliengo D, Gregori D, Fabricio AS, Gion M, Baldi I. Phenomapping of patients with primary breast cancer using machine learning-based unsupervised cluster analysis. Journal of Personalized Medicine. 2021 Apr 5;11(4):272. 33916398 10.3390/jpm11040272 PMC8067194

[58] Henriksen KL, Rasmussen BB, Lykkesfeldt AE, Møller S, Ejlertsen B, Mouridsen HT. Semi-quantitative scoring of potentially predictive markers for endocrine treatment of breast cancer: a comparison between whole sections and tissue microarrays. Journal of Clinical Pathology. 2007 Apr 1;60(4):397–404. 16775123 10.1136/jcp.2005.034447 PMC2001128

[59] Korsching E, Packeisen J, Agelopoulos K, Eisenacher M, Voss R, Isola J, Cytogenetic alterations and cytokeratin expression patterns in breast cancer: integrating a new model of breast differentiation into cytogenetic pathways of breast carcinogenesis. Laboratory Investigation. 2002 Nov;82(11):1525–33. 12429812 10.1097/01.lab.0000038508.86221.b3

[60] Ameh-Mensah C, Duduyemi BM, Bedu-Addo K, Atta Manu E, Opoku F, Titiloye N. The Analysis of bcl-2 in Association with p53 and Ki-67 in Triple Negative Breast Cancer and Other Molecular Subtypes in Ghana. Journal of Oncology. 2021 Jun 4;2021. 10.1155/2021/7054134 PMC8195641 34188682

[61] Li J, Chen Z, Su K, Zeng J. Clinicopathological classification and traditional prognostic indicators of breast cancer. International journal of Clinical and Experimental Pathology. 2015;8(7):8500–8505. 26339424 PMC4555752

[62] Van Laere SJ, Van den Eynden GG, Van der Auwera I, Vandenberghe M, van Dam P, Van Marck EA, Identification of cell-of-origin breast tumor subtypes in inflammatory breast cancer by gene expression profiling. Breast Cancer Research and Treatment. 2006 Feb;95:243–55. 16261404 10.1007/s10549-005-9015-9

[63] Zhou W, Jirström K, Amini RM, Fjällskog ML, Sollie T, Lindman H, Molecular subtypes in ductal carcinoma in situ of the breast and their relation to prognosis: a population-based cohort study. BMC Cancer. 2013 Dec;13(1):1–9. 24171825 10.1186/1471-2407-13-512 PMC4228470

[64] Parker JS, Mullins M, Cheang MC, Leung S, Voduc D, Vickery T, Supervised risk predictor of breast cancer based on intrinsic subtypes. Journal of Clinical Oncology. 2009 Mar 3;27(8):1160–7. 19204204 10.1200/JCO.2008.18.1370 PMC2667820

[65] Ihemelandu CU, Leffall LD Jr, Dewitty RL, Naab TJ, Mezghebe HM, Makambi KH, Molecular breast cancer subtypes in premenopausal and postmenopausal African-American women: age-specific prevalence and survival. Journal of Surgical Research. 2007 Nov 1;143(1):109–18. 17950079 10.1016/j.jss.2007.03.085

[66] Ihemelandu CU, Naab TJ, Mezghebe HM, Makambi KH, Siram SM, Leffall LD Jr, Basal cell–like (triple-negative) breast cancer, a predictor of distant metastasis in African American women. The American Journal of Surgery. 2008 Feb 1;195(2):153–8. 18083134 10.1016/j.amjsurg.2007.09.033

[67] Hu Z, Fan C, Oh DS, Marron JS, He X, Qaqish BF, The molecular portraits of breast tumors are conserved across microarray platforms. BMC Genomics. 2006 Dec;7(1):96. 16643655 10.1186/1471-2164-7-96 PMC1468408

[68] Prat A, Parker JS, Karginova O, Fan C, Livasy C, Herschkowitz JI, Phenotypic and molecular characterization of the claudin-low intrinsic subtype of breast cancer. Breast cancer research. 2010 Oct;12(5):R68. 20813035 10.1186/bcr2635 PMC3096954

[69] Prat A, Perou CM. Deconstructing the molecular portraits of breast cancer. Molecular Oncology. 2011 Feb 1;5(1):5–23. 21147047 10.1016/j.molonc.2010.11.003 PMC5528267

[70] Eom YH, Kim HS, Lee A, Song BJ, Chae BJ. BCL2 as a subtype-specific prognostic marker for breast cancer. Journal of Breast Cancer. 2016 Sep 1;19(3):252–60. 27721874 10.4048/jbc.2016.19.3.252 PMC5053309

[71] Paik S, Shak S, Tang G, Kim C, Baker J, Cronin M, A multigene assay to predict recurrence of tamoxifen-treated, node-negative breast cancer. New England Journal of Medicine. 2004 Dec 30;351(27):2817–26. 15591335 10.1056/NEJMoa041588

[72] Poikonen P, Sjöström J, Amini RM, Villman K, Ahlgren J, Blomqvist C. Cyclin A as a marker for prognosis and chemotherapy response in advanced breast cancer. British Journal of Cancer. 2005 Sep;93(5):515–9. 16091759 10.1038/sj.bjc.6602735 PMC2361595

[73] Soliman NA, Yussif SM. Ki-67 as a prognostic marker according to breast cancer molecular subtype. Cancer Biology & Medicine. 2016 Dec;13(4):496–504. 28154782 10.20892/j.issn.2095-3941.2016.0066 PMC5250608

[74] Ragab HM, Samy N, Afify M, Abd El Maksoud N, Shaaban HM. Assessment of Ki-67 as a potential biomarker in patients with breast cancer. Journal of Genetic Engineering and Biotechnology. 2018 Dec 1;16(2):479–84. 30733763 10.1016/j.jgeb.2018.03.002 PMC6353752

[75] Cascales HS, Burdova K, Middleton A, Kuzin V, Müllers E, Stoy H, Cyclin A2 localises in the cytoplasm at the S/G2 transition to activate PLK1. Life Science Alliance. 2021 Mar 1;4(3):e202000980. 33402344 10.26508/lsa.202000980 PMC7812317

[76] Kim SS, Alves MJ, Gygli P, Otero J, Lindert S. Identification of novel cyclin A2 binding site and nanomolar inhibitors of cyclin A2-CDK2 complex. Current Computer-Aided Drug Design. 2021 Feb 1;17(1):57–68. 31889491 10.2174/1573409916666191231113055 PMC7326642

[77] Prat A, Adamo B, Cheang MC, Anders CK, Carey LA, Perou CM. Molecular characterization of basal-like and non-basal-like triple-negative breast cancer. The Oncologist. 2013 Feb;18(2):123–33. 23404817 10.1634/theoncologist.2012-0397 PMC3579595

[78] Ahmed-Choudhury J, Agathanggelou A, Fenton SL, Ricketts C, Clark GJ, Maher ER, Transcriptional regulation of cyclin A2 by RASSF1A through the enhanced binding of p120E4F to the cyclin A2 promoter. Cancer Research. 2005 Apr 1;65(7):2690–7. 15805267 10.1158/0008-5472.CAN-04-3593

[79] Charan M, Verma AK, Hussain S, Misri S, Mishra S, Majumder S, Molecular and cellular factors associated with racial disparity in breast cancer. International Journal of Molecular Sciences. 2020 Aug 18;21(16):5936. 32824813 10.3390/ijms21165936 PMC7460595

